# Applied Mixed Generalized Additive Model to Assess the Effect of Temperature on the Incidence of Bacillary Dysentery and Its Forecast

**DOI:** 10.1371/journal.pone.0062122

**Published:** 2013-04-29

**Authors:** Weiping Ma, Xiaodong Sun, Yanyan Song, Fangfang Tao, Wei Feng, Yi He, Naiqing Zhao, Zhengan Yuan

**Affiliations:** 1 Department of Biostatistics and Social Medicine, School of Public Health, Fudan University, Shanghai, China; 2 Shanghai Municipal Center for Disease Control and Prevention, Shanghai, China; 3 Department of Biostatistics, College of Basic Medical Sciences, Jiao Tong University, Shanghai, China; Universidade Federal do Acre (Federal University of Acre), Brazil

## Abstract

**Background:**

Association between bacillary dysentery (BD) disease and temperature has been reported in some studies applying Poisson regression model, however the effect estimation might be biased due to the data autocorrelation. Furthermore the temperature effect distributed in the time of different lags has not been studied either. The purpose of this work was to obtaining the association between the BD counts and the climatic factors such as temperature in the form of the weighted averages, concerning the autocorrelation pattern of the model residuals, and to make short term predictions using the model. The data was collected in the city of Shanghai from 2004 to 2008.

**Methods:**

We used mixed generalized additive model (MGAM) to analyze data on bacillary dysentery, temperature and other covariates with autoregressive random effect. Short term predictions were made using MGAM with the moving average of the BD counts.

**Main Results:**

Our results showed that temperature was significant linearly associated with the logarithm of BD count for temperature in the range from 12°C to 22°C. Optimal weights in the temperature effect have been obtained, in which the one of 1-day-lag was close to 0, and the one of 2-days-lag was the maximum (p-value of the difference was less than 0.05). The predictive model was showing good fitness on the internal data with R^2^ value 0.875, and the good short term prediction effect on the external data with correlation coefficient to be 0.859.

**Conclusion:**

According to the model estimation, corresponding Risk Ratio to affect BD was close to 1.1 when temperature effect goes up for 1°C in the range from 12°C to 22°C. And the 1-day incubation period could be inferred from the model estimation. Good prediction has been made using the predictive MGAM.

## Introduction

Bacillary dysentery (BD) is the common intestinal infectious disease caused by Shigella, with fever, abdominal pain, diarrhea, tenesmus and mucus blood and pus as the main clinical symptoms, severe cases may have symptoms of systemic poisoning. The infection can be transmitted by the fecal-oral route via contaminated water, food, or person-to-person contact [1]. BD epidemics are frequent in overcrowded populations with inadequate sanitation and most cases occur in summer and autumn. Increased temperature and poor hygiene can affect the whole chain from food production to food on the table, including producing, processing, transport, preparation or storage and even in kitchen, allowing pathogens to multiply and leading to more patients of enteric infections [2]. BD is a notified communicable disease in China. In Shanghai the incidence of BD has significant seasonality throughout the year and is particularly high in the summer and autumn of recent years. Moreover the incidence of BD has been the highest among the Class A and B intestinal infectious diseases. BD has become the focus of the prevention and control of infectious diseases over the years. The effects of weather conditions, such as temperature, rainfall and relative humidity, on BD incidence have got much more concerning recently [3]–[6]. Generally in epidemiological studies, incidence is an important index which has become the focus in many researches. Incidence was calculated by dividing the disease counts with the total population. Since the study population was relatively stationary during the time period from 2004 to 2008 with the annual growth rate below 1%, (Table 1) the trend of incidence during that time period could be similarly prescribed by the trend of disease counts. Hence we used the BD counts as the response variable in our models.

In 2007, Ying Zhang used the seasonal autoregressive integrated moving average (SARIMA) models to discover that monthly maximum and minimum temperatures were significantly positively associated with dysentery transmission, and also applied the Hockey Stick model to find out that the thresholds for the effects of maximum and minimum temperatures were 17°C and 8°C respectively in the northern city, but no thresholds were detected in the southern city of China based on data from 1987 to 2000 [7]. In 2008, Ying Zhang also used the SARIMA models to quantify the relationship between temperature and BD in Jinan, a city of temperate climate northern China, controlling for the seasonality, lag time and long term time trend, suggesting that temperatures could become a predictor of the number of dysentery cases in temperate climate northern China [8]. The above studies used monthly and historical data to detect the association between temperatures and dysentery transmission, but weekly or more frequent data could make the estimation and prediction more accurate.

Reena B. K. Singh also reported that there was positive association between annual average temperature and the rate of diarrhea reports based on the data of 18 Pacific Island countries from 1986 to 1994, and the authors examined diarrhea notifications in Fiji in relation to estimates of temperature and rainfall, using Poisson log-linear regression analysis of monthly data for 1978–1998 in this study. The results showed that there existed positive association between extremes of rainfall and the rate of diarrhea reports [9]. Temperature was regarded as the key weather condition that affects the number of cases of BD in Chinese cities [7], [10]. Generalized additive model(GAM) with Poisson family distribution and splines were wildly used to analyze association between meteorology and mortality or incidence in some studies[11]–[13],

GAM is a probability model which requires the data to be independent among each individuals. But time series data are always autocorrelated, so that it is not proper to fit the time series data with GAM. Moreover, the temperature effect on the BD counts may be distributed in the days of different time lags, and this feature has never been involved in all the researches above.

Our study used daily BD cases and meteorological variables from 2004 to 2008 to fit mixed GAM (MGAM) with natural splines and to analyze the distribution of temperature effect in different delayed time on BD with controlling humidity and day-of-the-week (DOW) as confounding variables. The aims of this study were to investigate the impacts of temperature variations on BD counts in Shanghai and to make the dynamic forecasting to the BD counts in the external data.

## Materials and Methods

### Data

Shanghai is located in the eastern part of China and the city has a mild subtropical climate with four distinct seasons and abundant rainfalls. It is the most populous city in China comprising urban/suburban districts and counties, with a total area of 6,341 km2 and had a population of 13.9 million by the end of 2008. (Table 1) The data of the BD cases were from National Disease Supervision Information Management System, which is a Notifiable Diseases reporting system of real-time, online, based-on-case information. The cases were all clinical or laboratory-confirmed and reported by hospital diagnostic.

**Table 1:** Summary of Population in Shanghai.

Year	Population(million)	Population growth rate(%)
2004	13.52	
2005	13.60	0.582
2006	13.68	0.575
2007	13.79	0.788
2008	13.91	0.883

The daily meteorological data (including minimum, maximum, and mean temperature and relative humidity) were from the Shanghai Meteorological Bureau. The weather data were measured at a fixed-site station located in the Xuhui District of Shanghai.

The BD data and meteorological data were validated by an independent auditing team.

### Statistical Analysis

We used generalized additive model (GAM) and mixed GAM (MGAM)[14] with natural splines and logarithm link function to analyze BD counts with temperature, humidity and some other covariates in the fixed effect and autoregressive terms in random effect. The GAM was in the expression of (1),
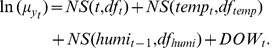
(1)


Here the conditional distribution of BD counts was assumed to follow a Poisson distribution approximately with mean 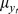 in the 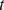
th day where 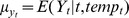,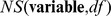 was a natural spline function, 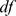 was the degree of freedom, 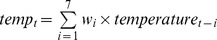 was the weighted average of the temperature within the week before time 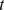 with 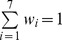,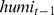 was the daily average of humidity at time 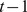, 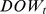 was dummy variable for day of the week.

Besides the weather condition, the BD counts are also affected by the demographic change, living status and some other factors varying with time. Some of the factors cannot be easily measured, then we applied natural spline function 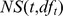 to explain the effect of those time-variant factors.

On the other hand, the data of the daily BD counts from 2004 to 2008 was a typical time series data and the data may autocorrelated, therefore we had the MGAM in the expression of (2)–(4).
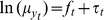
(2)

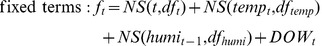
(3)

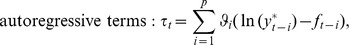
(4)where 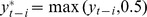, 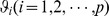 was the coefficient of autoregressive random effect. If additionally we set 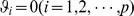, Model (2)–(4) could be expressed as (1), the generalized additive model (GAM). Those parameters (including the weights 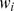
s) in both GAM and MGAM models were estimated by maximum partial likelihood method using Newton’s method.

Since the expectation of autoregressive term was 0, we could use the GAM model to determine the degree of freedom of the natural spline function for time trend 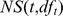, and rewrote model (1) as follow.
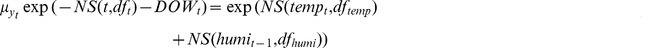
(5)


The right side of (5) was temperature effect, and the seasonal pattern of temperature was almost the same through different years. And the same pattern of the seasonality should occur on the left side. Thus we picked suitable degree of freedom 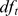 such that the seasonality of 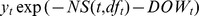 would be invariant through different years. When the effects of those factors changing with time on the BD counts were eliminated, the estimation on temperature effect could be unbiased. And the seasonality was demonstrated by the scatter plot. On the other hand, the selection of 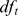 should be as small as possible to avoid underestimation of temperature effect on the BD counts.

If we increased the 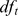 of the natural spline function, the smoothness of the approximation function would change a lot and the variation of the function would be hugely increasing simultaneously. Meanwhile the temperature effect and the humidity effect as the function of variable 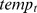 and 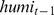 should be smooth enough. Therefore we selected the degrees of freedom of the natural spline functions on the condition that sharp peaks or valleys would not occur in the temperature and humidity effect curves, and the AIC (Akaike Information Criterion) was applied to find out the optimal one. We selected the order of autoregressive error term p such that the partial autocorrelation and autocorrelation could both fall in the set [−0.1, 0.1]. And the optimal one was also decided by the AIC criterion. To demonstrate the validity of the degree of freedom selection, we also fitted the data with 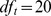
(4 per year), as it was described in other studies.

To make prediction, we need to modify the time effect in the model. The term 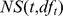 in (3) was replaced with 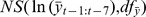, where 
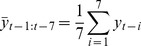
 was the moving average of BD counts in the past week. We selected the order of autoregressive residual term 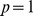 by ACF and PACF plot of the residuals. Here we had the predictive MGAM model (2), (6) and (7):

fixed terms:
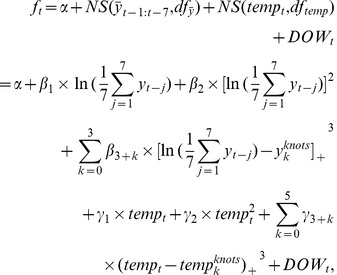
(6)

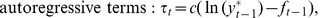
(7)where the degree of freedom 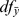
was determined by AIC criterion.

The parameters of the predictive model were also estimated by maximum partial likelihood estimator using Newton’s method.

All of the numerical analysis was conducted in the statistical software R (version 2.14). The GAM estimation was based on the results of the R package ‘GAM’, [15] and the MGAM estimation was based on the results of the R function ‘gamwithAR’. [16].

## Results

In our study the population was approximately 13.9 million residents (2008), and the number kept relative stable during our study period.

**Figure 1:**
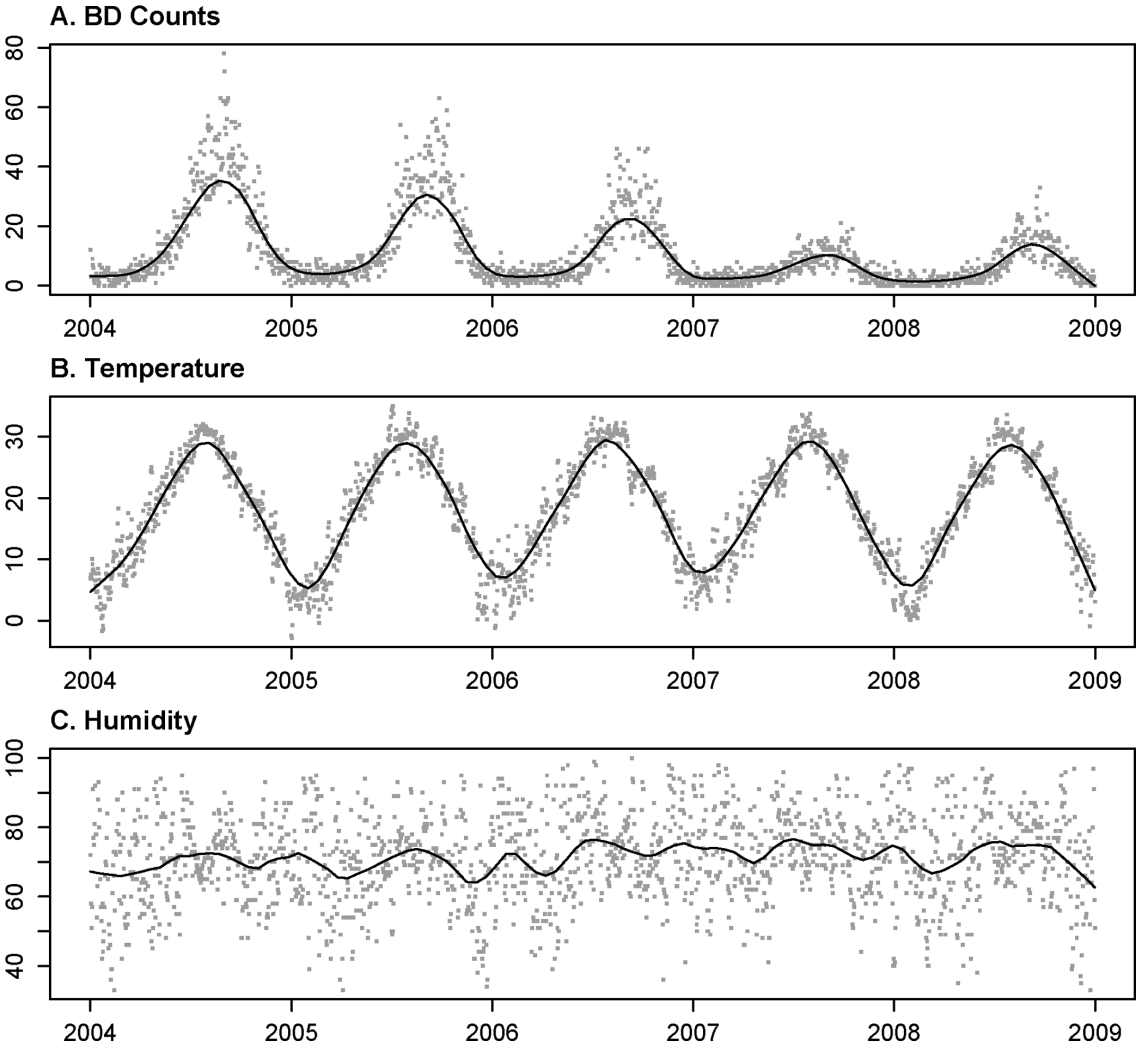
Scatter plot s of BD counts, Temperature and Humidity vs. Time. A. Daily BD counts from 2004 to 2008, with the solid line: Lowess estimation of BD counts. B. Daily averaged temperature from 2004 to 2008, with the solid line: Lowess estimation of averaged temperature. C. Daily averaged humidity from 2004 to 2008, with the solid line: Lowess estimation of averaged humidity.

There were total 20667 BD counts during the period from 2004 to 2008(1827 days), and the daily average was 11.31. Some more details were described in Table 2 and Figure 1A. During the period from 2004 to 2008, the range of 24 h mean temperature was from −2.8 to 35, and the range of 24 h mean humidity was from 33 to 100, the scatter plot of temperature and humidity were followed for demonstration (Figure 1B and Figure 1C).

**Table 2:** Summary of Daily BD Counts in Shanghai.

Year	Mean	sd	Q1	median	Q3	Min	Max
2004	18.45	16.64	5	11.5	30.75	0	78
2005	15.43	14.60	4	8	26	0	63
2006	11.10	10.76	3	6	17	0	46
2007	5.35	4.27	2	4	8	0	21
2008	6.26	6.25	2	4	9	0	33

**Figure 2:**
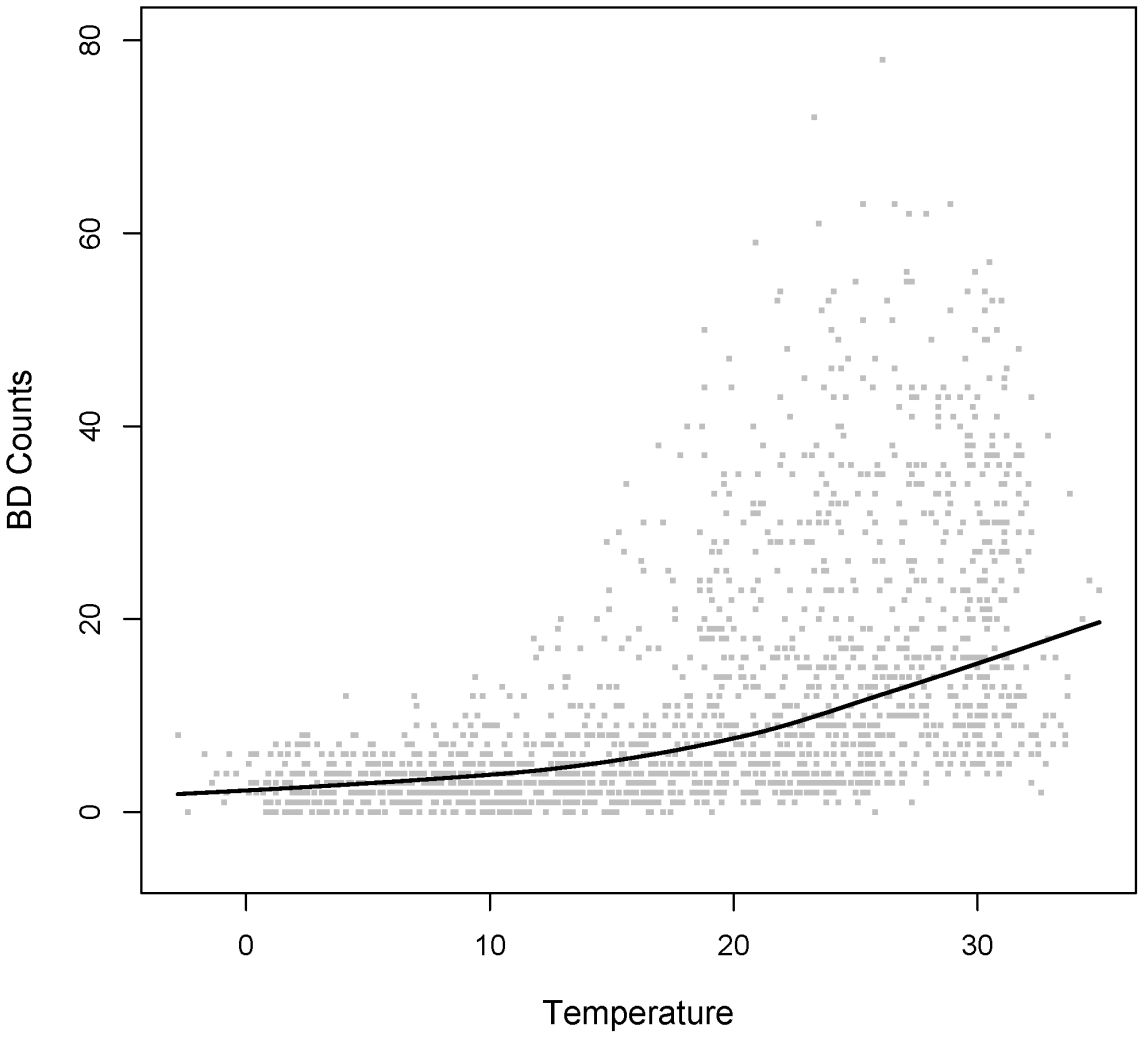
Scatter plot of BD counts vs. Temperature from 2004 to 2008. The solid black line is the Lowess estimator of the association between daily BD counts and daily average temperature.

**Figure 3:**
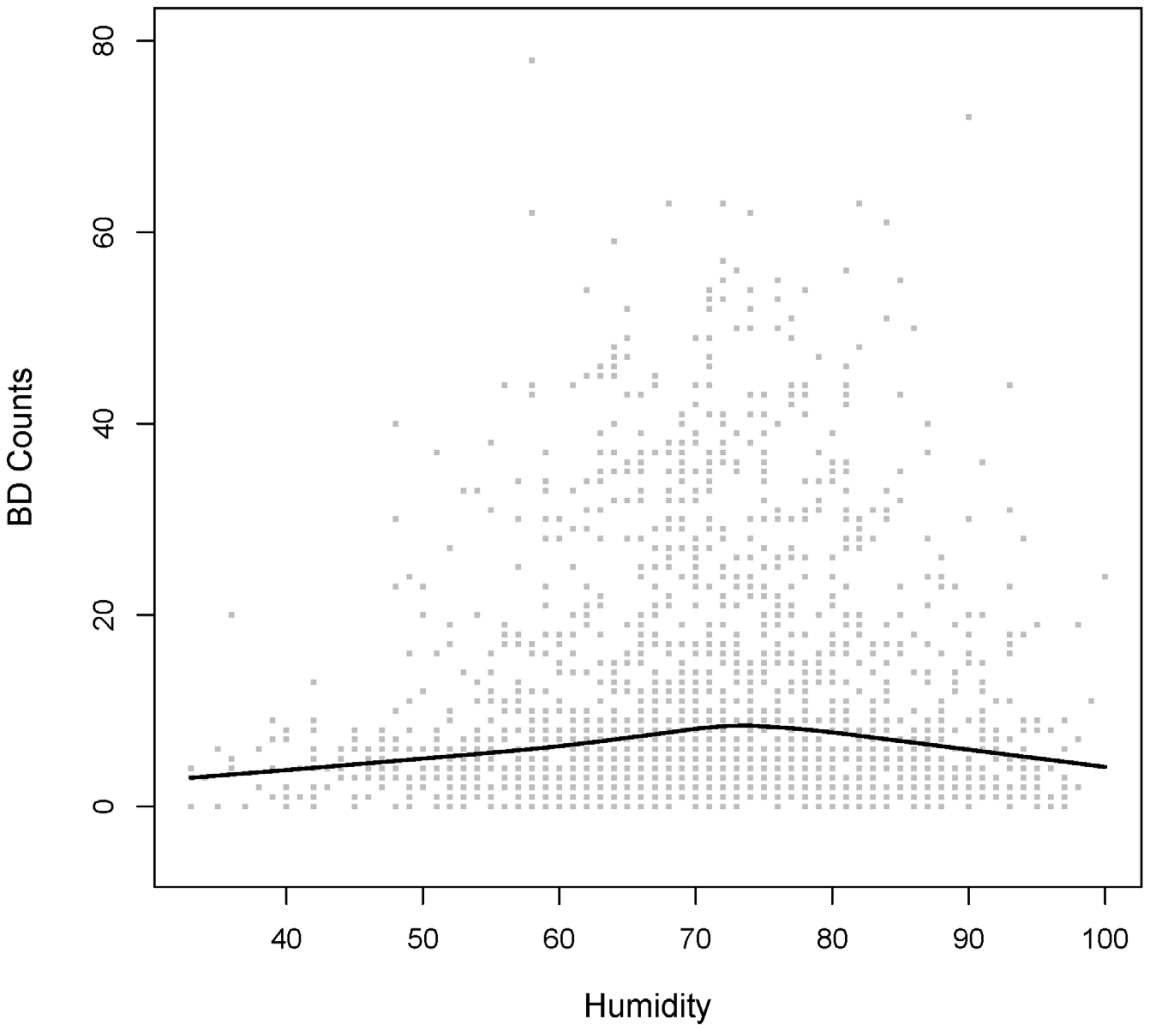
Scatter plot of BD counts vs. Humidity from 2004 to 2008. The solid black line is the Lowess estimator of the association between daily BD counts and daily averaged humidity.

From Figure 2 and Figure 3, we could roughly observe the strong statistical association between temperature and BD black line is the Lowess estimator of the association betweenever, there were only weak statistical associations between humidity and BD counts (Spearman correlation coefficient was 0.06) or temperature (Spearman correlation coefficient was 0.17).

**Figure 4:**
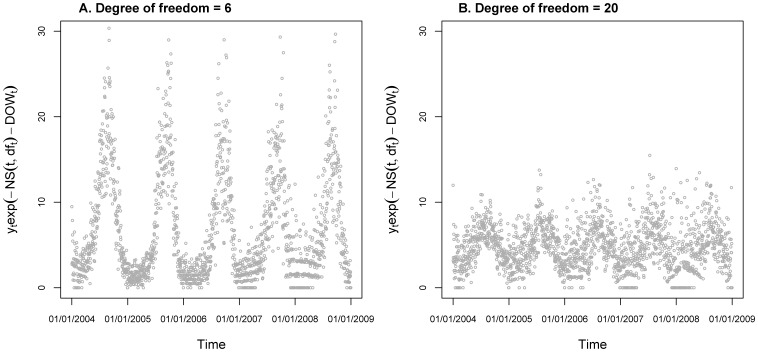
Scatter plot of y_t_exp(-NS(t,df_t_)-DOW_t_) vs. Time by GAM model. From expression (5), we shall assume that the term µ_t_exp(-NS(t,df_t_)-DOW_t_) will have stable seasonality which does not change in different years. To achieve this property we need to check the seasonality of the term y_t_exp(-NS(t,df_t_)-DOW_t_) by the scatter plot under the certain selection of the degree of freedom for the natural spline functions. The figure shows good seasonality with df_t_ = 6 in part A and week seasonality with df_t_ = 20 in part B.

**Figure 5:**
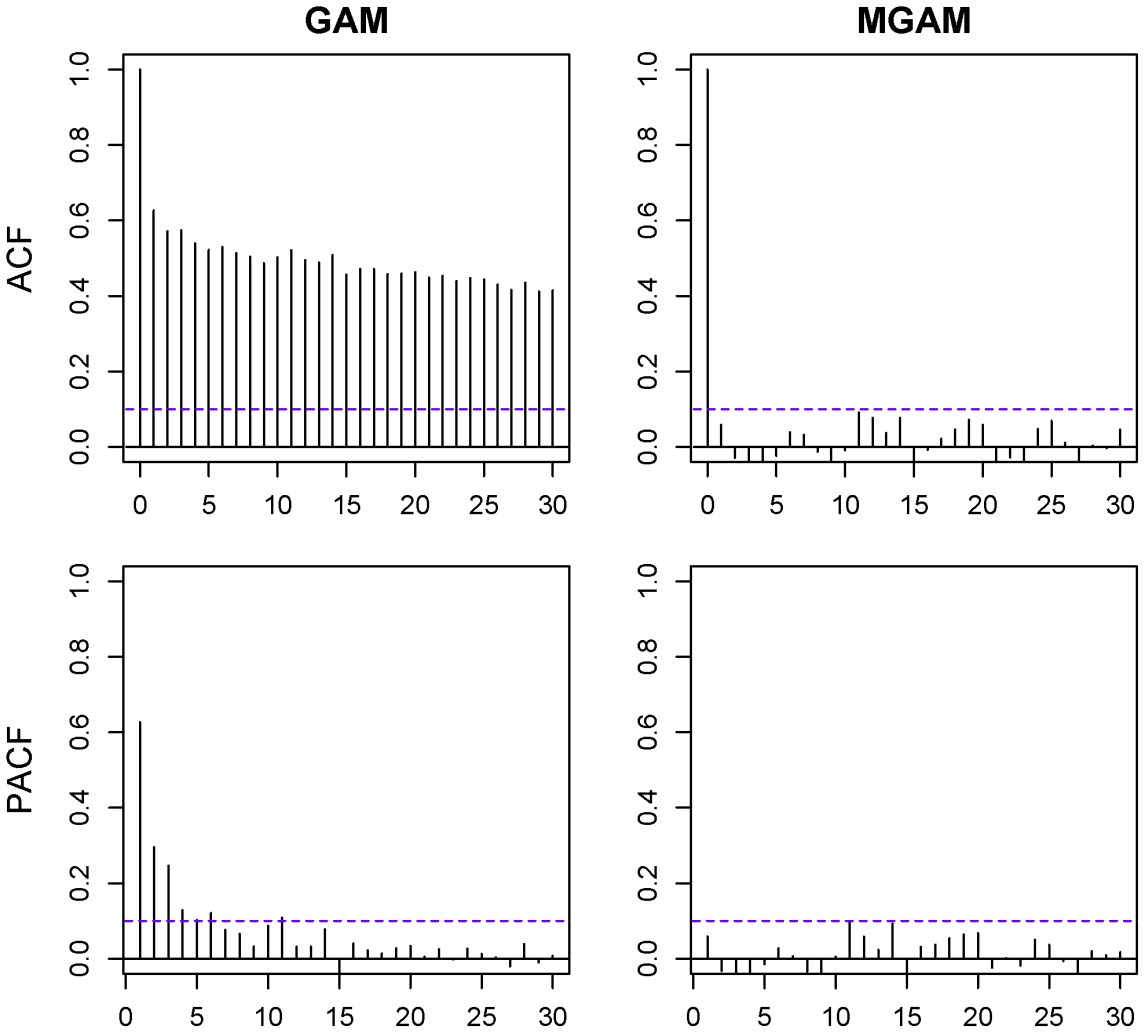
Residual autocorrelation and partial autocorrelation of GAM and MGAM with df_t_ = 6. Upper left is the autocorrelation function (ACF) of GAM residuals. Upper right is the ACF of MGAM residuals. Lower left is the partial autocorrelation function (PACF) of GAM residuals. Lower right is the PACF of MGAM residuals.

If we selected 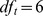 when fitting the data with GAM, the time trend curve 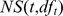
would be smooth enough and 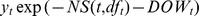 would show stable seasonality in the scatter plot against time (Figure 4A). Next we selected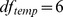 and 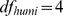. Then both temperature effect curve 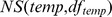 and humidity effect curve 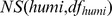 were relatively smooth, and AIC of the model reached its local maximum. However from Figure 5, the AC and PAC plots of GAM model were showing that the residual autocorrelation of GAM 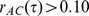 for some nonzero lag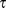, and also partial autocorrelation 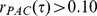 for some lags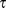. This implies that the GAM model was not suitable for the data. Therefore we used the MGAM instead. The temperature effect curve 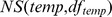 and humidity effect curve 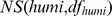 still kept smooth for 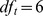, 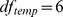 and 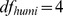, at the same time the AC and PAC plots of MGAM model were not showing obvious autocorrelation: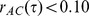 for all nonzero lags 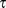 and the 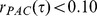 for all lags 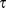
(Figure 5) by selecting the order of autocorrelation 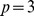. Therefore we could obtain the validity of the MGAM model for the BD counts data.

**Figure 6:**
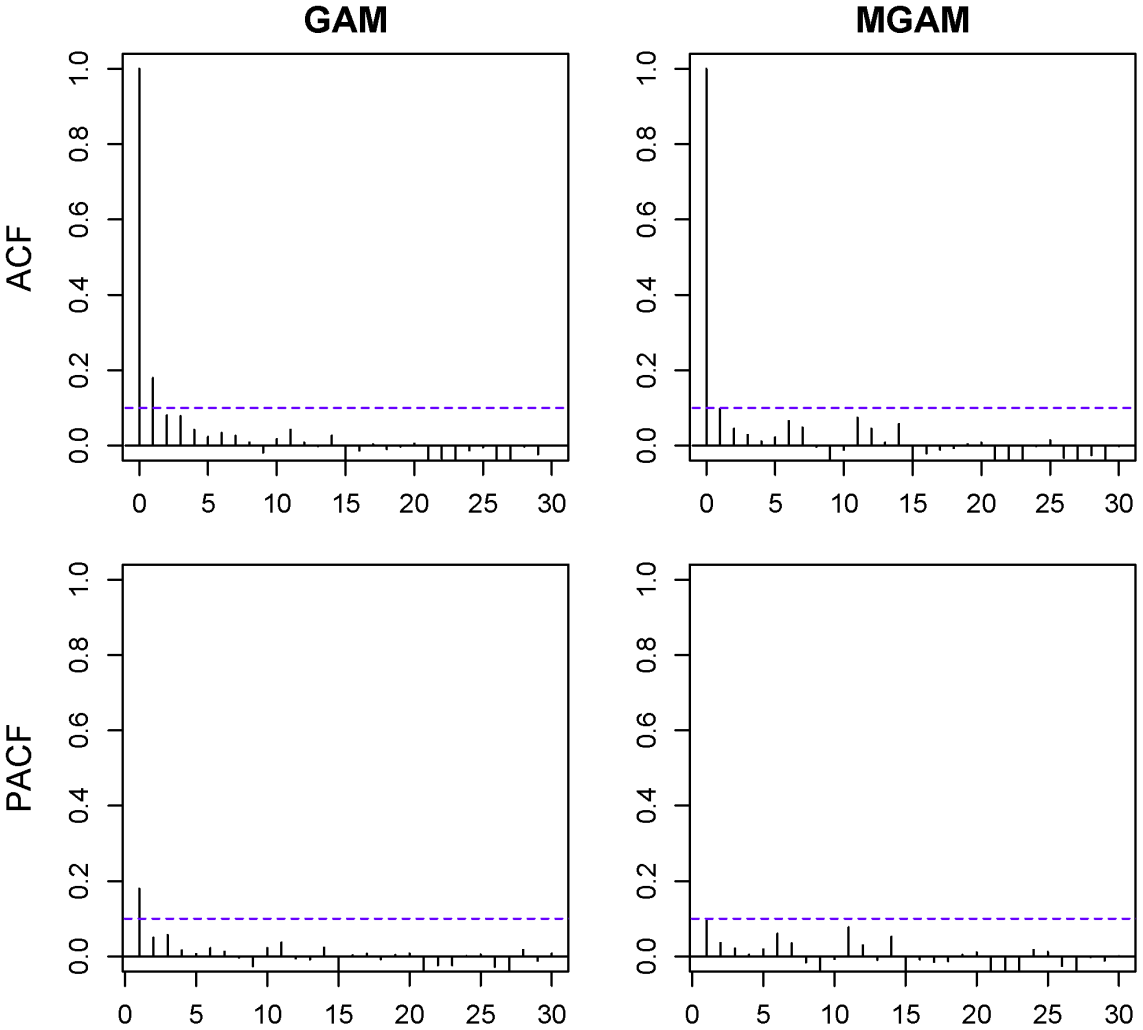
Residual autocorrelation and partial autocorrelation of GAM and MGAM with df_t_ = 20. Upper left is the autocorrelation function (ACF) of GAM residuals. Upper right is the ACF of MGAM residuals. Lower left is the partial autocorrelation function (PACF) of GAM residuals. Lower right is the PACF of MGAM residuals.

To make a contrast, we set 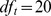 (4 per year) and used the same model to fit the data. We also demonstrated the seasonality of the term 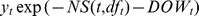 in a scatter plot (Figure 4B). From the figure we could see that the seasonality pattern was not very clear and the amplitudes of the terms were much lower than the corresponding ones in the figure of 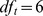 (Figure 4A). Meanwhile we also displayed the autocorrelation and partial autocorrelation of the residuals in Figure 6. We could observe that not only the AC and PAC plots of MGAM residuals were showing very week autocorrelation: 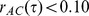 for all nonzero lags 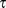 and the 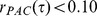 for all lags 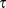, but also the ones of GAM residuals were almost having the same properties.

**Figure 7:**
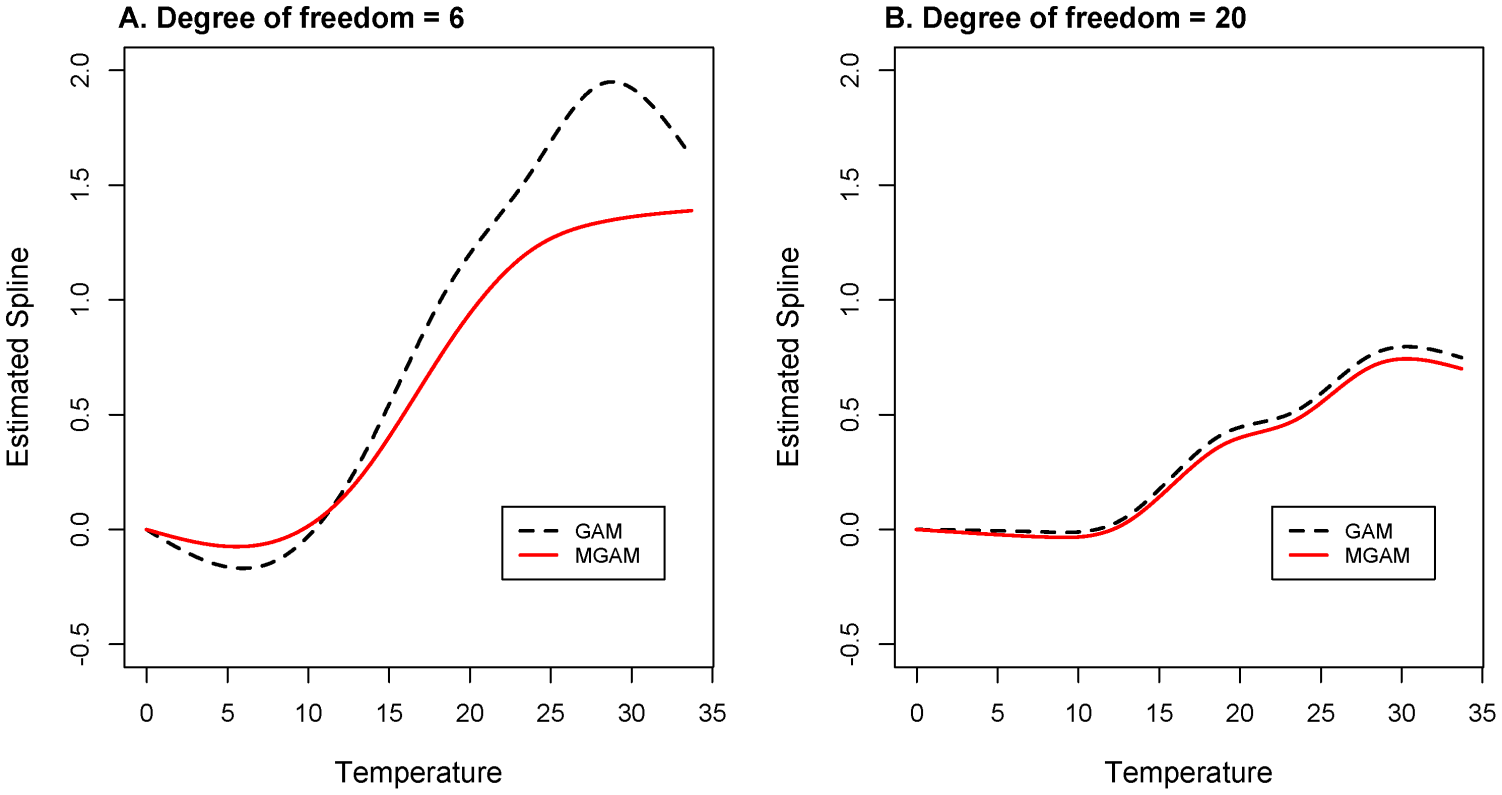
Estimated spline for temperature effect of MGAM and GAM. A. Temperature effect of MGAM and GAM estimated with df_t_ = 6. B. Temperature effect of MGAM and GAM estimated with df_t_ = 20. The red solid lines in both plots are the estimated spline for temperature effects on BD counts of MGAM model, and the black dashed lines are the estimated spline for temperature effects on BD counts of GAM model.

The estimated temperature effects of both GAM and Mixed GAM model were adjusted by the time trend and humidity effect. And the temperature effects curves were displayed in Figure 7. The estimated effect in the left part of the figure was based on the selection under AIC criterion when 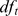 was determined as 6. In the temperature effect curve of Mixed GAM, we could find out that the curve was increasing with nearly linear trend (the slope was 0.1, corresponding Risk Ratio was 1.1) in the area from 12°C to 22°C, while the curve was almost flat in the area from 0°C to 12°C and from 22°C to 35°C. But the temperature effect curve of GAM was having the different pattern: increasing in the area from 7°C to 28°C, decreasing in the area from 0°C to 7°C and from 28°C to 35°C. While the temperature effect in the right part of the figure was estimated by setting 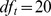. Under this circumstance we could see that the temperature term estimated by GAM and MGAM were quite close, but we could not find some clear linearity pattern in the curves.

**Figure 8:**
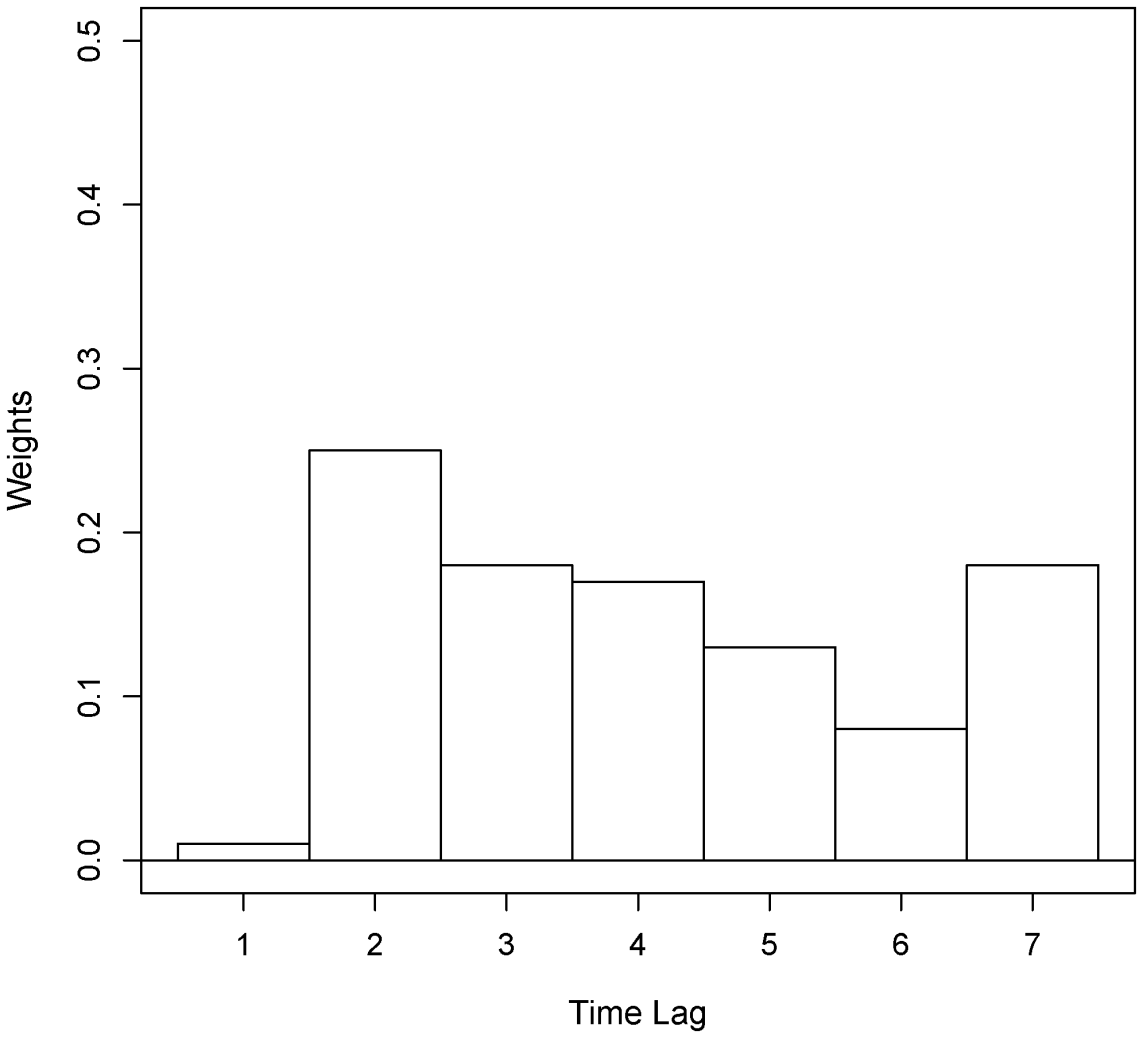
Maximum likelihood estimation on the temperature effect distribution of different time lags.

The temperature effect in model (1) was the weighted average of temperature from the day of lag 1 to the day of lag 7. The optimal weights were estimated as 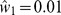, 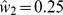, 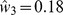, 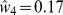, 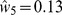, 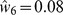, 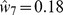. In Figure 8 We can find some specific patterns of the optimal weights estimation. The weight of 1-day-lag 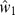 was the minimum close to 0, and the weight of 2-days-lag 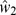 was the maximum. The difference between those two weights was statistically significant with p-value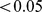. The other weights 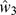,…,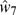 were not significantly different from each other. Therefore we could also assign equal weights 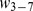
from lag 3 to lag 7. And the corresponding estimator 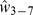 is 13.7%, which was significantly smaller than 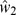.

**Figure 9:**
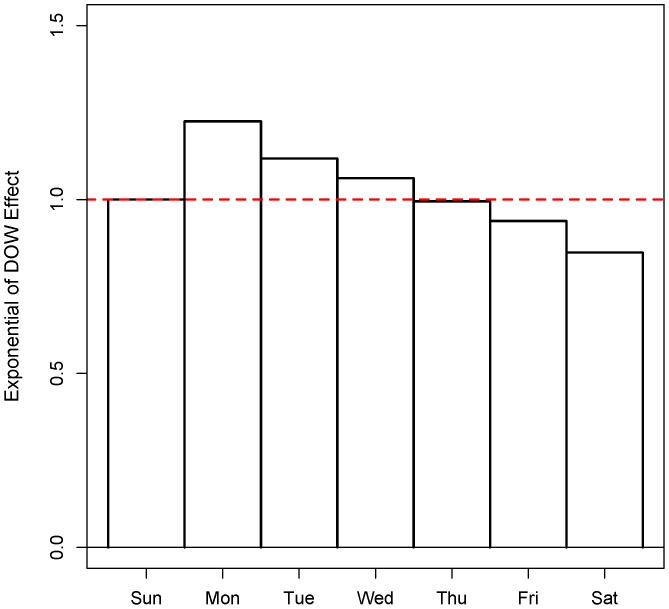
Exponential of Day-of-week effect estimated by MGAM.

In the figure of day of week (DOW) effect (Figure 9), we could observe that the maximum effect occurred on Monday and the minimum occurred on Saturday. Further, the effect followed a monotone decreasing trend from Monday to Saturday, but increasing on Sunday.

In the predictive model, we used 
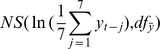
, to estimate the time effect instead of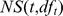, and degree of freedom 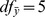 was also determined due to the smoothness of spline function. The estimated expression of predictive MGAM model was followed.
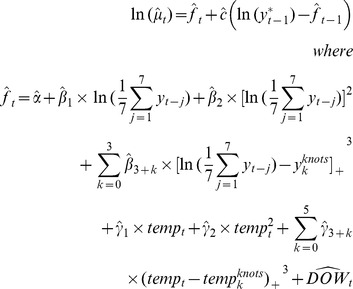



Where 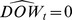 for *t* = Sunday, and 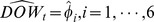 for *t* = Monday, Tuesday, … , Saturday.

**Figure 10:**
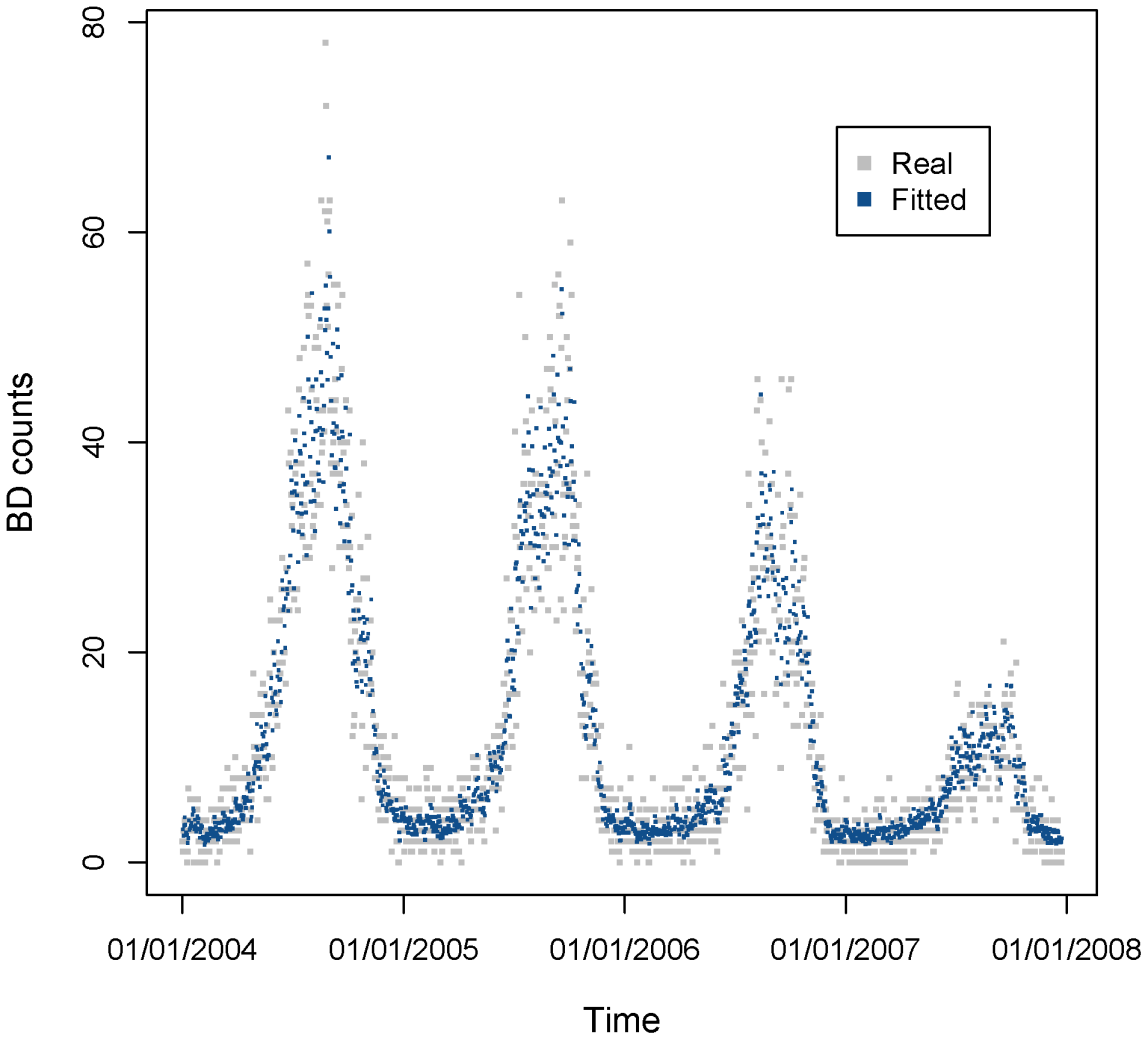
Real and fitted BD counts using the predictive MGAM model from 2004 to 2007. The light gray points are real daily BD counts from 2004 to 2007, and the dark blue points are fitted BD counts using predictive MGAM model. R-square of the model fitting is 0.875.

We fitted the predictive model (6) to the BD data in Shanghai from 2004 to 2007. Figure 10 showed the scatter plot of fitted values with the original BD counts. The figure demonstrated that the model fitted the real data well. And the R^2^ value of fitted and original data was 0.875, also showing good fitness.

**Figure 11:**
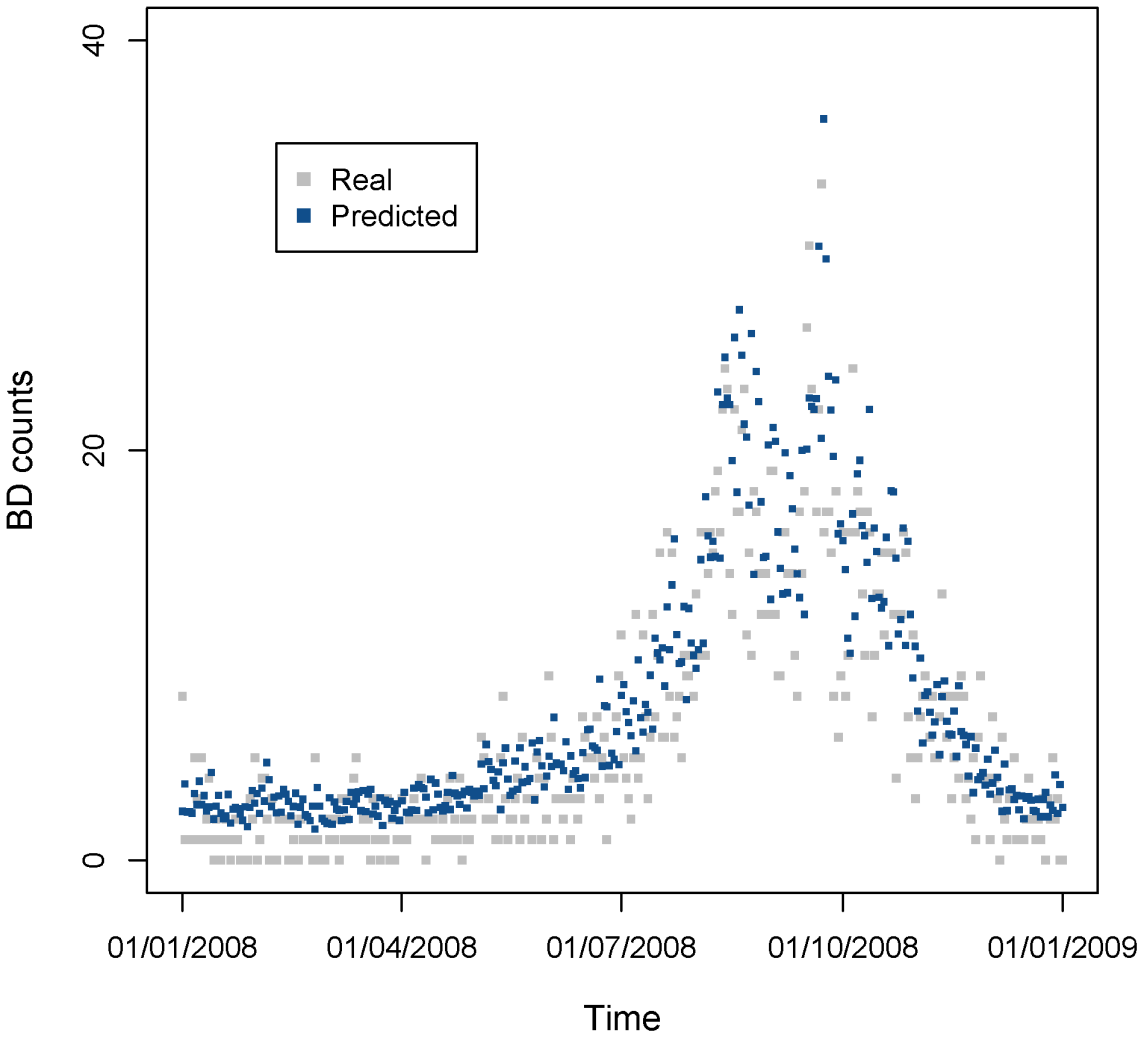
Real and predicted BD counts using the estimated expression of predictive MGAM model in 2008. The light gray points are real daily BD counts in 2007, and the dark blue points are predicted BD counts using predictive MGAM model. Correlation coefficient between the real and predicted value is 0.859.

To make prediction on the data in 2008, we estimated the parameters of model (7) (Table 3). Predicted values are displayed with the original BD counts in the scatter plots in Figure 11 which was showing the good prediction. And the correlation between predicted value and original counts was 0.859.

**Table 3:** Parameter estimation of predictive model.

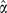	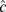	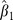	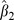	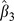	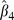	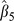	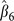
1.0480	0.1369	0.1462	0.00013	0.0756	−0.0886	−0.1144	0.1668
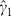	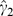	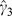	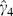	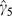	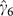	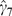	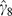
−0.0509	0.00011	0.00026	−0.00074	0.00052	−0.000045	−0.00022	0.00045
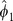	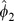	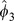	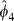	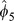	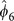		
0.2260	0.1323	0.0705	0.0078	−0.0378	−0.1538		
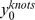	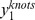	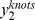	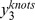				
0	1.2321	1.8827	2.9197				
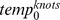	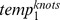	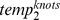	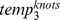	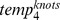	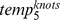		
0	8.1692	13.1517	18.9490	23.5810	28.1702		

## Discussion

### Model Fitting

In exploratory analysis we found out that the variation of BD counts was decreasing through different years. Then we selected the specific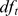 to make the scatter plot of 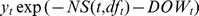 showing equal seasonality within each year. Therefore the variables with obvious and stable seasonality such as temperature effect could fit the term well. In the real case we selected 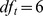 in the final models.

In some other environmental epidemiology studies, researchers often fitted the data of environmental and health conditions with GAM models to evaluate the effects of environment on human health [17]–[21]. And generally in studies of daily disease counts with GAM, degree of freedom of the natural spline function for the time trend was often selected to be 4 per year.

However, the health data and the environmental factors such as temperature were time series, which could be considered as the functions of time, thus the increasing of the degree of freedom 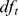 might lead to the overestimation of the time trend and hence the under estimation of the temperature effect. Additionally, since those time series data are autocorrelated to some extent, the autocorrelation patterns might not be captured by fitting with the GAM model.

To make the contrast we also selected the degree of freedom for the time trend to be 20 (4 per year). We could observe week seasonality of the term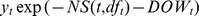 from its scatter plot against time *t*(Figure4B). Because the expectation of 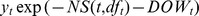 was 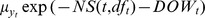, which equaled to 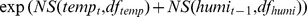 in the model expression, and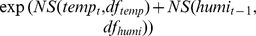 was a seasonal term showing the almost same pattern in each period(one year), it was not very appropriate to fit the term 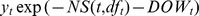 with a variable of strong and clear seasonality such as temperature. In another word, in the fitting procedure the temperature effect were explained by the time trend to some extent. On the other hand, the scatter plot showed that the amplitude of the term 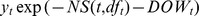 was much smaller than the term estimated with 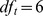 at most time point, which also implied the underestimation of the temperature effect.

The time trend natural spline functions 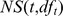 with degree of freedom 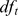 (usually chosen as 4 per year) in GAM were implemented also to make the AC and PAC of the GAM’s residuals bounded within [−0.1, 0.1]. [17], [19]–[21] However in this study, if we set 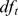 to be 4 per year, although the PAC and AC of the residuals might be well controlled (In Figure 6), the temperature effect on BD counts would decrease a lot. In fact, the implementation of the time trend spline was not aimed at reducing the autocorrelation but controlling the variation of time trend in order to keep the good seasonality of the environmental effects on the health conditions. Thus the unautocorrelated pattern of AC and PAC of the GAM’s residuals meant the over fitting of the time trend here.

Specific temperature effect structures could be obtained from the results. When temperature rises up above 22 C°, people start using air conditioning facilities and the indoor temperature will be relatively stable. Thus the increasing of outdoor temperature may not seriously affect citizen’s daily life, that is coincide with the flat part of the temperature effect curve estimated by MGAM over 22 C°(Figure 7A). Additionally, when temperature falls down below 12 C°, the activity of bacteria will fall down simultaneously. Prevalence of BD will decrease to a relatively low level under this circumstance, which means the infection of BD does not strongly depend on the change of temperature within the region below 12 C°. Between 12 C° and 22 C°, temperature was approximately linear correlated with the logarithm of BD counts, and the slope was positive. Different patterns in these regions could be demonstrated very clearly in the plot of temperature effect on BD counts estimated by MGAM, which is also regarded as the evidence verifying the conclusions of related studies [5], [10].

Moreover, if we look at the temperature effect estimated under the selection of 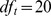 (Figure 7B), the temperature effect of GAM and MGAM were almost the same. This implies that even if the observations in the data were independent, the fitting MGAM would also capture the same temperature effect as GAM, and the conclusion has been verified through simulation studies. [16] But for dependent cases, the autocorrelation of the residuals might be shrunk a lot by the over fitting of time trend in GAM. Additionally, the temperature effect in the range from 15 C° to 35 C° was much smaller than the one estimated with 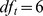 because of the over fitting of the time trend, which coincided with the amplitude decreasing of 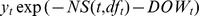 in Figure 4.

Consequently the model fitting results of GAM and MGAM showed that the selection 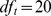 did not work well in our case. On the contrary we should use MGAM with the selection procedure proposed in the previous sections to fit the time series data of environmental effects on BD counts.

We also performed some sensitivity analysis to reveal the stability of the model estimation. We separately kicked out the first year observations, or the last year observations from the original data, and used the same model to practice estimation on both new data sets. The results showed that the selection of the degree of freedom for the natural spline functions were remaining the same. And the estimation of lagged temperature effect distributions were showing the similar patterns as the one of original estimation with the minimum at lag-1 day and the maximum at lag-2 day. This result coincided with our intuition that the lagged temperature effect should be a disease specified property which might not change over time.

### Prediction

Daily BD counts can be viewed as a time series data, which is usually be predicted by Autoregressive Integrated Moving Average (ARIMA) model in a short term. But ARIMA model could not take the information of the temperature and humidity into account to make prediction. Therefore we used the GAM based model in our study. There are also some other method to make the disease forecast. Method based on multiplicative Holt-Winters series, a general-purpose econometric method, [22] has still not considered the effect of some other covariates as environmental factors; machine learning based method such as SVM constructs linear or nonlinear patterns for the association between the input and output variables, [23] and only could predict the status of epidemic but not the specific case number; and generalized linear models (GLM) such as Poisson linear regression model, [11] which can be viewed as a simplification of GAM, could not capture the nonlinear pattern of the association between the environmental predictors and the disease counts. And those methods were not widely used. On the other side, as a probability model, GAM could only predict the BD counts in the way of using the conditional expectation. And the natural spline function of time trend 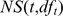 is estimated with the present data which cannot be used in prediction procedure. GAM is consequently not suitable for the short term prediction. Thus we considered MGAM model. There was also the time trend term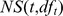 in the estimation expression of MGAM which must be modified in the predictive model. In real data analysis, we used the logarithm of 7-day moving average before the BD count day 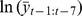 instead of the original time trend term. Obviously the predictive MGAM was the combination of probabilistic random effect model and the moving average model. The Pearson correlation between the original data and predicted data was 0.859 which was showing good prediction accuracy.

### Distribution of Delayed Temperature Effect

Since there are certain time delayed temperature effects on BD cases, and the delayed time may not be the same for all individuals, we should consider the distribution of delayed effect within a period before the BD count time. In this study we estimated the weights of temperature effect of 7 days before the BD count day. And the weights represented the estimated distribution of delayed temperature effect. We could infer that the incubation period of BD is about 1 day based on the fact that the weight of lag 1 was very close to 0 and the weight of lag 2 was the maximum. Theoretically we should consider the temperature delayed effect within longer period before the BD count day, but it would be hard to illustrate the incubation period by the time delayed effect on the period of excessive length, and we might easily observe the collinearity in the regression model which should be avoided. Those were the reason why we only used the time delayed effects within 7 days before the BD count day.

Significantly positive temperature effects on the BD counts have been reported in many previous studies, but the results of ours were more detailed and practical.

### Conclusions

Considering the autocorrelation of the data, the MGAM could model the association between the daily BD counts and the climatic factors such as temperature much better than GAM. We estimated the distribution of delayed temperature effects in the form of weighted average within the week before the BD count day. And we could also make good prediction with the MGAM based predictive model using the logarithm of 7-day moving average before the BD count day as the time trend effect.
